# Comparing the effect of group- based training along with text messaging and compact disc- based training on men’s knowledge and attitude about participation in perinatal care: a cluster randomized control trial

**DOI:** 10.1186/s12884-020-03471-0

**Published:** 2020-12-09

**Authors:** Vahideh Firouzan, Mahnaz Noroozi, Mojgan Mirghafourvand, Ziba Farajzadegan

**Affiliations:** 1 29 Bahman Hospital Research Center, Iranian Social Security Organization, Tabriz, Iran; 2 Department of Midwifery and Reproductive Health, School of Nursing and Midwifery, Isfahan University of Medical Sciences, Isfahan, Iran; 3 Social Determinants of Health Research Center, Tabriz University of Medical Sciences, Tabriz, Iran; 4 Department of Community Medicine, Medicine School, Isfahan University of Medical Sciences, Isfahan, Iran

**Keywords:** Group-based training, Compact disk-based training, Perinatal care, Participation, Knowledge, Attitude, Men

## Abstract

**Background:**

Men’s participation in perinatal care is one of the key factors in promoting maternal and neonatal health. The effects of various methods of training on men’s knowledge and attitude about participation in perinatal care can be different. So, this study aimed to compare the effect of two methods of training on men’s knowledge and attitude about participation in perinatal care.

**Methods:**

This cluster randomized control trial was conducted in three midwifery clinics in Tabriz, Iran between May and August 2018. Each clinic was randomly assigned to intervention (group- based training along with text messaging and CD- based training) and control groups. Seventy-five men were enrolled in three groups and evaluated for their knowledge and attitude about participation in perinatal care. Before and 3 months after the intervention, a researcher-made questionnaire was completed by the participants. Data were analyzed using descriptive and inferential statistics (paired t-test, one-way ANOVA, ANCOVA, chi-square, Kruskal-Wallis and Fisher exact tests).

**Results:**

The mean (SD) score of men^,^ s knowledge and attitude about participation in perinatal care had a significant increase in group- based training along with text messaging after the intervention compared to the score of before the intervention (*p* < 0.001, *p* = 0.005, respectively), but the mean (SD) score of men^,^ s knowledge and attitude had not a significant increase in CD- based training and control group after the intervention compared to the score of before the intervention. The mean (SD) score of men^,^s knowledge and attitude about participation in perinatal care in group- based training along with text messaging were significantly higher than in CD- based training (*p* < 0.001, *p* = 0.039, respectively) and control group (*p* = 0.001, *p* = 0.021, respectively) after the intervention, respectively. However, the mean (SD) score of men^,^ s knowledge and attitude in CD- based training were not significantly different from the control group after the intervention.

**Conclusion:**

Group- based training along with text messaging was more effective in improving the knowledge and attitude of men about participation in perinatal care compared to CD- based training. So, its implication in educational programs for the men is recommended.

**Trial registration:**

IRCT, IRCT20160224026756N4. Registered 27 May 2018.

## Background

Men’s participation in perinatal care is one of the key factors in promoting maternal and neonatal health [1, 2]. In most parts of the world, men in the family are responsible for important choices about allocating financial resources and caring behaviors which directly affect the health of women and infants [3]. Although it has been found that men’s behavior affects the reproductive health of their wives and infants, most maternal and child health programs have focused on women’s participation and education, with men being ignored. Men do not have access to information that helps them make informed decisions and improve their wives’ health [4]. According to studies, various factors influence men’s participation in perinatal care. These factors are at different levels including community, health care system, family, and individual level [5–8]. These include perceptions, beliefs, and attitudes toward women’s health as a feminine task, leading to poor male participation in women’s reproductive health [2, 6–11]. Further, some of the known individual barriers including men’s education level, income, and limited awareness of men’s role in reproductive health, hinder this partnership [1, 5–8]. A study to illuminate expectant first-time fathers’ experiences of participation during pregnancy in three Nordic countries (Denmark, Finland and Sweden) showed that fathers wanted to participate and be responsible from the beginning of pregnancy [12]. The men’s need to education to enhance participation in prenatal, delivery, and postnatal care as well as its positive effects on maternal and neonatal health has been shown in numerous studies in different countries [1, 13–18]. The results of a study in rural areas of Pakistan showed that training men to increase their participation in perinatal care would improve some maternal and neonatal health indicators [19]. Adeleye and Okonkwo showed that trained men were more likely to recognize a danger sign of pregnancy and delivery [20]. Also, a study in Bangladesh showed a significant relationship between men’s education and their active participation in perinatal care [21]. In addition, the results of a study showed that men’s participation in prenatal counseling sessions increased their engagement in postpartum care, duration of exclusive breastfeeding, use of contraceptive methods, improving couples’ relationships and shared decision making [22].

Following the Cairo international conference on population and development (ICPD) in 1994, the positive role of men has been emphasized to promote reproductive health as well as to achieve the millennium development goals (MDGs) including maternal health, gender equality, reducing child mortality, eradicating illiteracy and diseases [23]. In Iran, no policies or programs exist that encourage participation of men in the perinatal period. In recent years, because of efforts to encourage women to have vaginal delivery, “delivery preparation classes” have been conducted for pregnant women including one session in which the men participate. Practically, however, this is executed at a limited number of health centers and its execution is not problem-free [2]. The effects of various methods of training on men’s knowledge and attitude about participation in perinatal care can be different. Group education is a method for stimulate thinking, challenging the beliefs and training inter-personal skills and if the learners would be ready for participating in the discussion the education would have an acceptable success [24]. Another training method is teaching through compact disks (CDs) which would be applied by transferring the concepts using texts, audio, images and video, more simply, extensive, and attractive [25]. Since no study has been conducted to compare the effect of different methods of training on men’s knowledge and attitude about participation in perinatal care in Iran, therefore, this study aimed to determine and compare the effect of two methods of group- based training along with text messaging and compact disc (CD) - based training, on men’s knowledge and attitude about participation in perinatal care.

## Methods

CONSORT guidelines were adhered for reporting of this trial.

### Study design

This study was a cluster randomized control trial with three groups (intervention 1, intervention 2 and control groups). The population of this study includes husbands of pregnant women (with gestational age of 20–22 weeks) who referred to three midwifery clinics affiliated to the Social Security Organization of Tabriz city, East Azerbaijan province, Iran from May to August 2018. There were a large number of pregnant women and their husbands with the same socio-economic status who referred to these clinics. Also, it was easier to access pregnant women and their husbands in these places. In this study to determine how many samples are needed to create a good chance of finding an effect (if the effect really exists) and considering that the minimum recommended value for the test power is 80%, we considered a test power of 95%. Based on the confidence interval of 95%, a test power of 95% (using G-power software), and considering a sample loss rate of 10%, sample size of 24 was estimated for each group.

### The inclusion and exclusion criteria

The inclusion criteria were husbands of pregnant women with gestational age of 20–22 weeks, lack of participation of men in any educational intervention or training about men’s participation in perinatal care, living with their wife, and no problem in relationships between couples (by asking participants). The exclusion criteria were unwillingness to continue to cooperate at any stage of the research and lack of full participation of men in the training sessions held in group- based training along with text messaging.

### Procedures

**Fig. 1:**
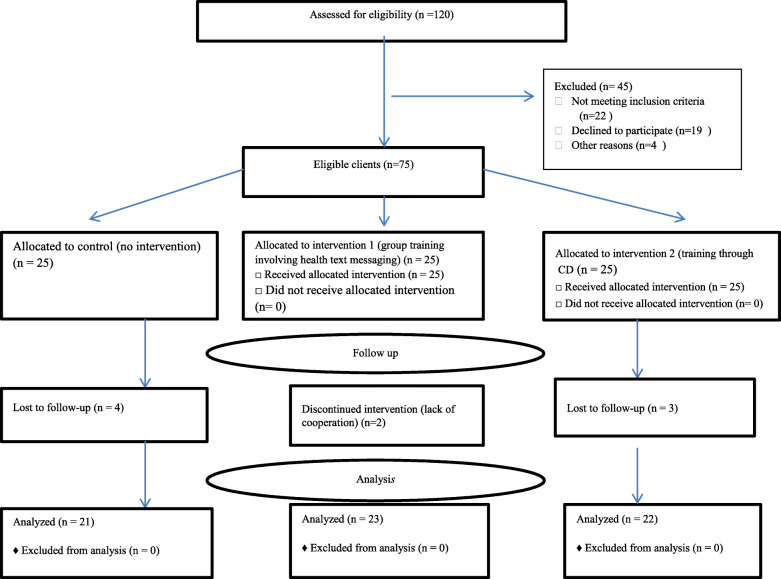
Trial flowchart

In the present study for preventing interactions and relationships between participating men about the interventions, the three midwifery clinics were randomly assigned to intervention (group- based training along with text messaging and CD- based training (and control groups by first author (VF). In this regard, one of the midwifery clinics was selected for group- based training along with text messaging, another clinic for CD- based training group and third clinic for control group. In the midwifery clinics, using convenience sampling, the medical files of 120 pregnant women were evaluated and participants who had the inclusion criteria were selected (Fig. 1). Then, after making phone calls to them, other inclusion criteria were asked and the time for their husband visit was settled. After visiting 75 eligible men and assuring them of the confidentiality of their information, written informed consent was obtained from all the participants (Fig. 1). The researcher performed the group- based training using different training methods (speech, displaying images, sharing the members’ experiences, and question and answer), board and computer in two sessions each lasting for 2 h with one-week interval on Fridays. At the end of the second session, the men were notified to receive health messages via mobile at their desired time. From the day after the second group education session, three to four health text messaging were sent weekly (about the participation of men in perinatal care) up to 3 months after the intervention. After the second group training session, the researcher distributed a CD among men of the CD-based training group about men participation in perinatal care (contents were similar to those provided at group-based training). This CD consisted of the training text, questions, images, and voices. Training content included changes in women’s body during pregnancy and the role of men in women’s adaption to these changes; common complaints during pregnancy and men’s role in women adaption to them; the men’s participatory role in perinatal care; the father’s relationship with the fetus and the mother; preparation for childbirth and fatherhood; danger signs during pregnancy and how to deal with them; the role of men in delivery process; social support and its consequences during pregnancy, childbirth and postpartum; The role of men in providing social support for pregnant women, during childbirth and postpartum; psychological support for pregnant women and in postpartum period; postpartum grief and men’s supportive role; postpartum depression and the role of men; barriers to men’s participation during pregnancy, childbirth, and postpartum and its remedies. Valid scientific books, articles, and websites were applied for preparation of the training contents [2, 26–31]. In this study, no action was taken for the control group.

### Primary outcomes

The primary outcomes included men^,^ s knowledge and attitude about their participation in perinatal care that were measured by a researcher-made questionnaire. The content of questions on men’ s knowledge and attitude about their participation in perinatal care were based on training content. This questionnaire was completed by the participants at two stages (before the intervention and 3 months after the intervention). The aforesaid questionnaire contained three sections: demographic characteristics, knowledge measurement, and attitude measurement. Knowledge measurement tool consisted of 28 multiple choice questions and the score of one was assigned to each “correct” answer. For “incorrect” and “not sure” answers, a score of zero was considered. The minimum score of zero and the maximum score of 28 were considered for the knowledge measurement tool. An example of a knowledge assessment question was: “When a man talks to his pregnant wife about her worries, it reduces her anxiety”. Attitude measurement tool had 23 questions with 5-point Likert scale (totally agreed, agreed, I have no idea, disagreed, totally disagreed). The assigned scores for measuring negative attitude were: 0 for totally agreed, 1 for agreed, 2 for I have no idea, 3 for disagreed and 4 for totally disagreed. Furthermore, the assigned scores for measuring positive attitude were: 4 for totally agreed, 3 for agreed, 2 for I have no idea, 1 for disagreed and 0 for totally disagreed. The minimum score of zero and the maximum score of 92 were considered for the attitude measurement tool. An example of an attitude assessment question was: “The duty of a man during pregnancy and postpartum is just work outside the home and earning an income”.

Content validity method was used to approve the validity of the data collection tools and test-retest method was applied for its reliability. Cronbach’s alpha coefficients for knowledge and attitude questionnaires were 0.71 and 0.89, respectively. The intra-cluster correlation coefficients (ICC) for knowledge and attitude questionnaires were 0.88 (95% CI = 0.59–0.96) and 0.98 (95% CI = 0.94–0.99), respectively that indicated the stability of the questionnaires.

### Statistical analysis

Statistical analysis of the present study was conducted using SPSS 23 software. The Chi-square, one-way ANOVA, Kruskal-Wallis and Fisher’s exact tests were used for assessing the consistency of the three groups in terms of socio-demographic characteristics. To compare mean score of men^,^ s knowledge and attitude about participation in perinatal care in each group paired t-test was used before and after the intervention. To compare the mean score of men^,^s knowledge and attitude about participation in perinatal care between the three groups one-way ANOVA was used before the intervention. To compare the mean score of men^,^s knowledge and attitude about participation in perinatal care between the three groups ANCOVA test was used after the intervention. The significance level for statistical tests was considered less than 0.05 (*P* < 0.05).

### Ethical considerations

This study was registered in the registry for clinical trials (IRCT20160224026756N4). The Ethics Committee of Isfahan University of Medical Sciences approved the protocol of this study (code number: IR.MUI. Rec.1395.3.599) and written informed consent, anonymity, confidentiality and the right of leaving the research at any desired time were preserved.

## Results

The results showed that the mean age of the men and their wives, number of children, frequency distribution of the job of men and their wives, the educational level of the men and their wives, family type, fetal sex and couples’ tendency to pregnancy had no significant difference between the three groups (*p* >  0.05) (Table 1).

**Table 1:** Comparison of socio-demographic characteristics of the participants between the three groups

Variable	Group- based training along with text messaging *n* = 23 number (percentage)	CD- based training *n* = 22 number (percentage)	Control group *n* = 21 number (percentage)	*P*-value*
Age (years)				
›21	0 (0)	1 (4.5)	1 (4.8)	
26–30	5 (21.7)	7 (31.9)	4 (19)	
31–35	12 (52.1)	8 (36.4)	11 (52.4)	0.837
36–40	3 (13.1)	4 (18.1)	4 (19)	
41–45	3 (13.1)	2 (9.1)	1 (4.8)	
Spouse age (years)				
›21	1 (4.3)	2 (9.1)	1 (4.8)	
21–25	6 (26.1)	3 (13.6)	2 (9.6)	
26–30	10 (43.4)	10 (45.5)	6 (28.6)	0.48
31–35	3 (13.1)	4 (18.2)	10 (47.6)	
36–40	3 (13.1)	3 (13.6)	2 (9.6)	
Number of children				
0	17 (73.9)	15 (68.2)	14 (66.7)	
1	5 (21.7)	5 (22.7)	5 (23.8)	0.077
2	1 (4.3)	2 (9.3)	2 (9.5)	
Education level				
Elementary	2 (8.7)	2 (9.1)	1 (4.8)	
Intermediate	1 (4.3)	2 (9.1)	2 (9.5)	
Highschool	4 (17.4)	4 (18.2)	4 (19)	0.807
Diploma	7 (30.4)	6 (27.3)	8 (38.1)	
Academic	9 (39.1)	8 (36.4)	6 (28.6)	
Job				
Employee	3 (13)	6 (27.3)	6 (28.6)	
Worker	7 (30.4)	6 (27.3)	4 (19)	0.556
Freelancer	7 (30.4)	8 (36.4)	9 (42.9)	
Other	6 (26.1)	2 (9.1)	2 (9.5)	
Spouse education level				
Elementary	1 (4.3)	1 (4.5)	0(0)	
Intermediate	2 (8.7)	2 (9.1)	1 (4.8)	0.781
Highschool	2 (8.7)	0 (0)	2 (9.5)	
Diploma	5 (21.7)	6 (27.3)	8 (38.1)	
Academic	13 (56.5)	13 (59.1)	10 (47.6)	
Spouse job				
Housewife	17 (73.9)	15 (68.2)	14 (66.7)	0.961
Employee	4 (17.4)	4 (18.2)	5 (23.8)	
Other	2 (8.7)	3 (13.6)	2 (9.5)	
Family type				
Living with spouse and children	20 (87)	21 (95.5)	18 (85.7)	
Living with spouse, children and other people	3 (13)	1 (4.5)	3 (14.3)	0.611
Fetal sex				
Girl	15 (65.2)	1 (59.1)	11 (52.4)	0.705
Boy	8 (34.8)	9 (40.9)	10 (47.6)	
Couples tendency to pregnancy				
Male	0	1 (4.6)	4 (19)	
Female	4 (17.4)	3 (13.6)	4 (19)	0.166
Both	17 (73.9)	18 (81.8)	13 (62)	
Non	2 (8.7)	0	0	

* *P* < 0.05 was considered significant

### Primary outcomes

The results showed that the mean (SD) score of men^,^ s knowledge and attitude about participation in perinatal care were not significantly different between the three groups before the intervention (*p* = 0.944, *p* = 0.228, respectively). The results showed that the mean (SD) score of men^,^ s knowledge (*p* < 0.001) and attitude (*p* = 0.005) about participation in perinatal care had a significant increase in group- based training along with text messaging after the intervention compared to the score of before the intervention, but the mean score of men^,^ s knowledge and attitude had not a significant increase in CD- based training and control group after the intervention compared to the score of before the intervention (Table 2). Also, the mean (SD) score of men^,^s knowledge (*P* < 0.001) and attitude (*P* = 0.011) about participation in perinatal care had a significant difference between the three groups after the intervention (Table 3).

**Table 2:** Comparing the mean scores of men^,^ s knowledge and attitude before and after the intervention in each group

Variable	Group	Before the intervention Mean (SD)	After the intervention Mean (SD)	Statistical test result	
				t	***P***-value*
Knowledge	**Group- based training along with text messaging**	18.6 (3.4)	23.3 (2.0)	−7.41	< 0.001
	**CD- based training**	19 (4.5)	20.22 (4.08)	−1.93	0.067
	**Control group**	18.6 (3.5)	18.6 (3.4)	1/000	0.329
Attitude	**Group- based training along with text messaging**	64.2 (9.0)	68.7 (9.5)	−3.13	0.005
	**CD- based training**	66.3 (13.50)	67 (11.24)	−0.70	0.488
	**Control group**	69.4 (8.7)	69 (8.6)	1.36	0.186

* *P* < 0.05 was considered significant

**Table 3:** Comparing the mean scores of men^,^ s knowledge and attitude after the intervention between the three groups

Variable	Group- based training along with text messaging Mean (SD)	CD- based training Mean (SD)	Control group Mean (SD)	Statistical test result	
				F	***P***-value*
**Knowledge**	23.3 (2.0)	20.2 (4.0)	18.6(3.4)	28.79	001/0>
**Attitude**	68.7 (9.5)	67.0 (11.2)	69.0 (8.6)	4.82	011/0

* *P* < 0.05 was considered significant

The results showed that the mean (SD) score of men^,^s knowledge and attitude about participation in perinatal care in group- based training along with text messaging were significantly higher than in CD- based training (*p* < 0.001, *p* = 0.039, respectively) and control group (*p* = 0.001, *p* = 0.021, respectively) after the intervention, respectively. However, the mean (SD) score of men^,^ s knowledge and attitude in CD- based training were not significantly different from the control group (Table 4).

**Table 4:** Pairwise comparison in terms of the mean scores of men^,^ s knowledge and attitude after the intervention

Variable	Group- based training along with text messaging to the control group		CD- based training to the control group		Group- based training along with text messaging to the CD- based training	
	Mean difference (%95 CI)	***P***-value	Mean difference (%95 CI)	***P***-value	Mean difference (%95 CI)	***P***-value*
**Knowledge**	4.6 (3.1–6.2)	> 0.001	1.4 (−0.19–2.9)	0.103	3/3 (1.7–4.8)	> 0.001
**Attitude**	3.8 (0.5–7.3)	0.021	0.4 (−2.9–3.8)	0.986	3.4 (0.13–6.7)	039/0

* *P* < 0.05 was considered significant

## Discussion

This study was conducted to compare the effect of two methods of group- based training along with text messaging and compact disc (CD)- based training on men’s knowledge and attitude about participation in perinatal care. The results of the present study showed that the mean score of men^,^ s knowledge and attitude about participation in perinatal care had a significant increase in group- based training along with text messaging after the intervention compared to the score of before the intervention. Although no study has been conducted on educational intervention with a combination of group- based training and text messaging and its effect on men’s knowledge and attitude about perinatal care in Iran, some studies over the impact of men’s education on their knowledge and attitude about participation in perinatal care have suggested that educational interventions have made positive changes in men’s knowledge and attitude, and promoted maternal and neonatal health [32–34]. In this regard, Alawode et al. in Nigeria found increased knowledge and improved performance of men during pregnancy through mobile phone texting [35]. Also, Allport et al. believed that innovative ways to reach father involvement in children’s lives are currently under investigation, including use of mobile technologies show promise in effectively engaging fathers and improving family health [36]. Kerstis et al. in Sweden showed that participating in father groups (parental classes) might help convince fathers to take more parental leave and build stronger relationships with their partner and child [37].

Adeleye and Okonkwo indicated a significant increase in knowledge and a meaningful change in attitude of men about the role of fathers in preventing maternal death through education via lectures and leaflets [20]. Researchers in a study in Bangladesh found that there was a significant relationship between active male participation in perinatal care and receiving information through TV, posters, billboards and healthcare providers [21]. Mullany et al. in a clinical trial in Nepal found that male education along with their wives promoted men’s preparation for childbirth (including planning to transfer the pregnant women to the hospital at delivery time, planning for emergencies and etc.) [38].

The results of the present study showed that the mean score of men^,^s knowledge and attitude about participation in perinatal care in group- based training along with text messaging were significantly higher than in CD- based training after the intervention. However, the mean score of men^,^ s knowledge and attitude in CD- based training were not significantly different from the control group. It seems that the reason for the lack of significant changes in the mean scores of men’s knowledge and attitude in CD- based training group is due to the lack of sufficient opportunity to make adequate use of the educational content of the CD due to men’s busy. In this regard, the findings of the present study revealed that the majority of participating men were the sole source of family income and their wives in most cases were housewives. Meanwhile, the disadvantages of CD include lack of face-to-face communication between the trainer and learner, no exchange of information and experiences between group members as well as between the trainer and the learner, which in turn causes less motivation [39]. Baldwin et al. showed that fathers wanted more guidance and support around the preparation for fatherhood, and partner relationship changes [40]. Also, Sarkadi et al. concluded that there is enough support to urge both professionals and policy makers to improve circumstances for involved fathering [41]. Based on the results of the present study, group- based training along with text messaging could be used as an appropriate method to improve men’s knowledge and attitude about participation in perinatal care.

### Strengths and limitations

One of the strengths of this study is the implementation of educational intervention on men’s knowledge and attitude about participation in perinatal care, for the first time, and almost all the principles of clinical trial, were observed to prevent selection bias. In the present study, coordinating with participating men to attend clinics for trial registration and also attend sessions in group- based training required frequent phone calls, which was time consuming. In this regard, 20 days before the intervention, participating men were repeatedly contacted and invited to register. Also, reminder text messages were sent to attend the training sessions on time.

One of the limitations of this research is individual differences between participating men in learning educational materials; therefore, this may affect data collection. The results of this trial may not be generalizable, because other settings, with different participants, and differing extents of training or motivation of healthcare providers could produce different results.

## Conclusion

Based on the results, group- based training along with text messaging was more effective in improving the knowledge and attitude of men about participation in perinatal care compared to CD- based training. It is worth noting that educational interventions should be designed and implemented in accordance with the real life conditions of people. Although, in the present study the educational content was the same in both training methods, participating men did not adequate use the educational content of the CDs and as a result, the use of CD- based training was not associated with positive results in promoting men’s knowledge and attitude about perinatal care. It seems that despite the small number of participants in this study, holding group- based training classes on holidays and in the men’s leisure time, has provided a great opportunity for them to learn and exchange ideas, followed by sending health text messages to continue learning and thus improve their knowledge and attitude about participation in perinatal care. Therefore, group- based training along with text messaging could be used as an appropriate method by policymakers and health planners in designing effective, culture-based educational interventions in men in the workplace, health centers and childbirth preparation classes.

## Data Availability

The datasets generated and/or analysed during the current research are not publicly available as individual privacy could be compromised but are available from the corresponding author on reasonable request.

## Abbreviations

ICPD: International conference on population and development
MDGs: Millennium development goals
CD: Compact disc
ICC: Intra-cluster correlation coefficient
ANCOVA: Analysis of covariance
ANOVA: Analysis of variance
CI: Confidence interval
